# Research Trends on Pillared Interlayered Clays (PILCs) Used as Catalysts in Environmental and Chemical Processes: Bibliometric Analysis

**DOI:** 10.1155/2022/5728678

**Published:** 2022-03-02

**Authors:** Iván F. Macías-Quiroga, Julián A. Rengifo-Herrera, Sandra M. Arredondo-López, Alexander Marín-Flórez, Nancy R. Sanabria-González

**Affiliations:** ^1^Department of Chemical Engineering, Universidad Nacional de Colombia sede Manizales, Campus La Nubia, km 7 vía al Aeropuerto, AA 127, Manizales, Colombia; ^2^Centro de Investigación y Desarrollo en Ciencias Aplicadas “Dr. Jorge J. Ronco” (CINDECA) (CCT-La Plata CONICET, UNLP, CICPBA), Departamento de Química, Facultad de Ciencias Exactas, Universidad Nacional de La Plata, 47 No. 257, La Plata 1900, Argentina; ^3^Unit of Strategic Information Analysis, Library Section, Universidad Nacional de Colombia sede Manizales, Campus La Nubia, km 7 vía al Aeropuerto, AA 127, Manizales, Colombia

## Abstract

Over the last four decades, a large number of studies have been published on pillared interlayered clays (PILCs) used as adsorbent materials and catalysts or supports for transition metals in heterogeneous catalysis. Particularly, PILCs have been used for water treatment through advanced oxidation processes (AOPs) to remove organic pollutants. They have also been studied in various chemical and environmental processes. Because of the growing interest in PILCs, this article is focused on analyzing scientific publications such as research/review articles and book chapters from the last four decades (from 1980 to 2019) through a bibliometric analysis (BA) to visualize and describe research trends on PILCs. By narrowing the bibliographic search to titles, keywords, and abstracts of publications related to PILCs, using Scopus and Web of Science (WoS) (the two scientific databases), a total of 3425 documents have been retrieved. The bibliometric dataset was analyzed by VantagePoint^®^. The main research trends identified in the last four decades were the use of PILCs in environmental processes (34.4% of total publications) along with chemical processes (petrochemical reactions 17.5%, SCR NOx 10.8%, and decomposition 8.2%). In environmental processes, PILCs have been used in photo-oxidation (32%), CWPO (21.1%), and heterogeneous catalysis (19.4%). Phenols, dyes, and VOCs have been the main pollutants studied using PILCs as catalysts. Fe, Ti, Zr, Cu, and Co are the most supported active phases in PILCs. Other research trends grouped by characterization techniques, countries, research areas, institutes, scientific journals that have published the most on this topic, number of publications per 5-year period, and most frequently used keywords through the last four decades have been identified. It was determined that the number of publications on PILCs has increased since 1980 and the countries with the highest number of publications are China, Spain, and The United States of America.

## 1. Introduction

The amount of wastewater generated and its pollution load are constantly growing worldwide [1]. Therefore, wastewater treatment is one of the main challenges that humanity will face in the coming decades [2]. Biological, physical, and chemical techniques have been used for wastewater treatment. Nevertheless, some of the conventional methods (e.g., filtration, adsorption, and coagulation-flocculation) for wastewater treatment do not degrade pollutants but simply transfer contaminants from one phase to another [3]. Furthermore, several of these pollutants are recalcitrant to biological degradation and toxic to microorganisms. In recent years, advanced oxidation processes (AOPs) have been studied for the removal of a wide number of organic contaminants to increase wastewater biodegradability as a pretreatment prior to a subsequent biological treatment [4, 5]. Versatility of AOPs is further boosted by the fact that they may generate •OH radicals in a variety of ways [3, 6, 7]. Currently, the three AOPs that have been most widely studied are heterogeneous photocatalysis (TiO_2_), photo-Fenton process, and H_2_O_2_/UV [4]. However, among AOPs, the catalytic wet peroxide oxidation (CWPO) process is considered a low-cost technology for the removal of pollutants, overcoming the drawbacks of homogeneous processes [4, 8].

CWPO allows the removal of recalcitrant organic compounds under mild conditions when using hydrogen peroxide (H_2_O_2_) and a solid catalyst with redox properties to generate •OH from the H_2_O_2_ decomposition [9]. Different types of catalysts have been used in CWPO processes, including alumina, zeolites, functionalized carbonaceous supports, and PILCs [10]. Pillared clays have been used as active solid materials in photocatalysis, CWPO, catalytic wet air oxidation (CWAO), and Fenton-like processes [11].

Although clays are very useful for several applications in the field of catalysis, the lack of permanent porosity is their main disadvantage [12]. To avoid this drawback, a way to expand the clay layers was found by inserting stable “pillars” in the interlayer space, increasing the surface area of the material and creating a high pore volume [12, 13].

By the end of 1970s and beginning of 1980s, the terms “pillared” and “pillaring” were coined [13, 14]. It was found that robust inorganic molecules could be intercalated into the clay interlayer space by a cationic-exchange mechanism, followed by solvent removal [13–15]. Aluminum is the main element used to synthesize pillars in PILCs because it can form Keggin-type polycations [AlO_4_Al_12_(OH)_24_(H_2_O)_12_]^7+^ that are incorporated into the interlayer space to form rigid aluminum oxide pillars after calcination [16, 17]. These materials show a greater surface area, pore volume, and improved thermal and mechanical stability (depending on the type of pillar inserted) compared to unmodified clays [18, 19]. Particularly, clay-based catalysts have generated interest in petroleum refineries and chemical and pharmaceutical industries [20–22]. Recently, PILCs have been used in environmental catalysis [23], pollution remediation [24], enzyme immobilization [25], biosensor development [26], adsorption processes for wastewater treatment [27], and storage/slow release of nitric oxide (NO) [28]. Thus, PILCs have a wide variety of barely explored applications [20].

Bibliometric analyses (BAs) allow the study of large volumes of scientific metadata (bibliographic records) to determine the evolution and emerging areas in a research field, based on scientific literature data from databases [29]. BAs identify global trends and future directions and provide an overview of large amounts of scientific papers [30–32], whereas review articles examine, summarize, and discuss previous studies of a research topic to assess challenges and advances in the area of interest [33, 34] through distinct approaches [35]. Moreover, BAs analyze the impact and relevance of publications, authors, countries, institutes (universities), and scientific journals associated with a specific theme [36].

In this context, the objective of this study was a bibliometric analysis on research trends in environmental and chemical processes utilizing pillared clays. In particular, this article shows the results of a systematic review of catalytic processes that used PILCs over the last four decades, focusing on those addressed to the reduction of water pollutants. Furthermore, clay minerals used on PILC synthesis, type of active phase supported, and characterization techniques of these materials were identified. Apart from these research trends, countries, research areas, institutes, and scientific journals that have published the most on this topic, the number of publications per 5-year period, and most frequently used keywords have also been included.

## 2. Materials and Methods

The methodology adopted for this study followed the procedure used by Macias-Quiroga et al. in a previous bibliometric study about research trends on the advanced oxidation process [4]. This study considered literature published during the last four decades from January 1, 1980, to December 31, 2019. Data collection was conducted between October 5 and 7, 2020. Scopus and Web of Science (WoS) databases were used as metadata sources; these databases contain 95% of worldwide publications [37].

The queries used to retrieve information from both databases are summarized in Table 1. Data were retrieved and analyzed using VantagePoint^®^ (version 13.1, Search Technology, Inc.) by text data mining. Since data formats retrieved from Scopus and Web of Science (WoS) are different, a manual verification was carried out over each unit of analysis. Likewise, the computer tool “List Cleanup,” of VantagePoint^®^ software, was used. The right combination and cleaning of data was mandatory for the proper treatment of bibliometric data, thus preventing bias and errors in the meta-data processing.

Data obtained from each database (Scopus and WoS) were combined, homogenized, and cleaned, identifying double-counting data (duplicates). It was necessary to manually rename some entries of analysis that were similar in both databases but differently abbreviated. After a process of standardization and deletion of homonym authors and institutions through a text data mining process, 2511 documents were obtained for bibliometric analysis (Table 1). To classify the scholarly impact of researchers, the *h*-index was used. The index quantifies the scholarly impact regarding the number of papers co-authored by the researcher with at least *h* citation search [39, 40] and it has been widely employed to measure the productivity and citation impact [41].

Because “KeyWords Plus” are equally effective as authors Keywords in BA, they were included in this study [4, 42]. They were both merged in a group named “Combined Keywords.” To eliminate the influence of “KeyWords Plus,” entries were analyzed by the number of records.

Concerning limitations and scope, this BA was unable to determine whether the number of citations by authors was the result of self-citations or even positive/negative citations. Similarly, it was not possible to specify each author's contributive weight in the articles through this bibliometric analysis. As a result, each author was considered to have the same contribution in publications [4].

## 3. Results and Discussion

**Figure 1:**
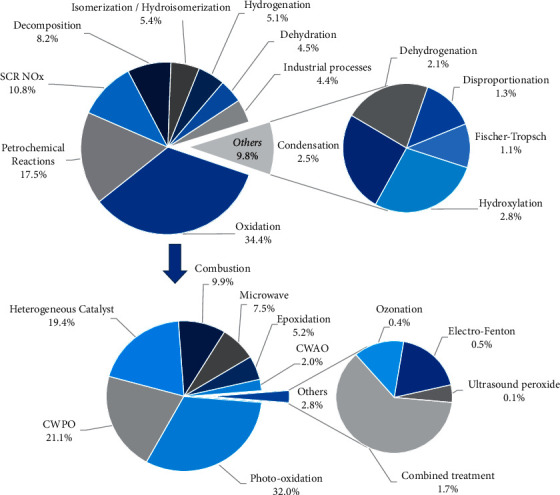
Distribution (%) of chemical and environmental processes that use pillared clays as catalysts over the past 40 years.

Different environmental and chemical processes that have used pillared clays as catalysts are summarized in Figure 1. Among the different applications of PILCs, the three main processes that have used them are oxidation techniques (34.3%), petrochemical reactions (17.5%), and selective catalytic reduction (SCR) of NOx (10.8%)[43]. Likewise, Figure 1 shows the variety of applications of PILCs in catalytic reactions such as isomerization, hydrogenation, hydroxylation, dehydrogenation, among others. This versatility of pillared clays in various catalytic processes is attributable to the high acidic properties (surface acidity), porous structure, relatively good thermal stability, and catalytic active species/metal oxide pillars of PILCs [18, 43].

Oil industry has used catalysts based on clays such as kaolinites, saponites, beidellites, and montmorillonites in catalytic cracking processes [44, 45]. Clay minerals subjected to acid treatments or pillarization processes have been used in the conversion of heavy hydrocarbons into light ones in oil refining [44–46]. Iron-pillared clays are selective for the pyrolysis heavy gas oil (HGO) and high-density polyethylene (HDPE), converting them into light hydrocarbons (C_10_ to C_23_) [46]. Similarly, Al- and Al-Fe-PILCs have been used in the catalytic cracking of medium-density polyethylene, providing higher yields of liquid products in the C_15_–C_20_ range, with lower costs than zeolites used for this type of processes [45]. PILCs have also been used for cracking vegetable oils in the production of biofuels [47]. In the last four decades, the percentage of publications on PILCs as catalysts in petrochemical reactions are distributed as follows: cracking/hydrocracking (37%), alkylation (23%), desulfurization/hydrodesulfurization (12%), pyrolysis (9%), hydroconversion (5%), hydration (5%), oligomerization (5%), fluid catalytic cracking FCC (3%), and coking (1%).

Until late 1990s, most research performed on the synthesis and application of pillared clays focused on petroleum cracking and refining [18, 48]. However, in the last 5 years, PILCs have not only been used in reactions such as alkylation, dehydrogenation, hydrocracking, and isomerization (Figure 1), but also in processes such as SCR/NOx and the complete oxidation of volatile organic compounds (VOCs), where PILCs have been used as metal catalyst supports [43]. Since the 2000s, the application of PILCs in environmental processes has become more frequent [11, 49–51], especially in the treatment of pollutants in gas or liquid phases through advanced oxidation processes, as displayed in Figure 1. In particular, photo-oxidation and CWPO processes have been extensively studied in the treatment of pollutants in water [3, 10, 11, 50].

**Figure 2:**
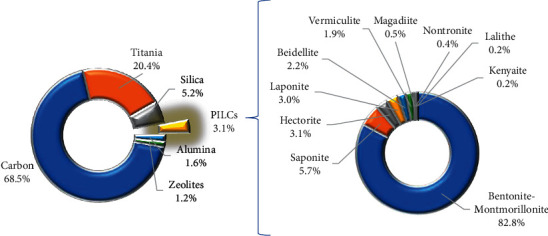
Catalytic supports used in AOPs and clay minerals used as precursors of PILCs.

Figure 2 shows the catalysts and/or catalytic supports used in AOPs. It can be seen that the three mostly used catalytic supports in advanced oxidation processes are carbon-based catalysts (68.5%), titanium oxides (20.4%), and silica (5.2%). PILCs represent 3.1% of the materials used as supports for the active phases in catalytic oxidations. The five most commonly used natural swelling clay minerals and synthetic smectites as precursors for the synthesis of PILCs are montmorillonite (82.8%), saponites (5.7%), hectorites (3.1%), laponites (3.0%), and beidellites (2.2%). Montmorillonites (MMT), the main component of bentonites, are typically used in pillaring processes. MMT are clay minerals 2 : 1 layer consisting of two Si tetrahedral sheets separated by one Al octahedral sheet (T-O-T) [52]. Isomorphic substitutions in the sheets create deficits of positive electric charges [53]. These negative charges are compensated by exchangeable cations in the interlayer space, allowing a modification of the material via pillarization. That is why bentonites have been widely used as catalytic supports for active phases in AOPs [17].

Different porous materials (including PILCs) have been used as catalytic supports to overcome the drawbacks of homogeneous catalysis in AOPs (Figure 2). For instance, materials such as zeolites, pillared clays, silica, silicates, and carbons have been utilized to support iron in Fenton-type processes for wastewater treatment [54].

The catalytic activity of TiO_2_-pillared clays has been tested in photo-oxidation processes for degradation of pollutants, with promising results since these materials combine the adsorption capacity of clays and the photocatalytic ability of TiO_2_ [55–57]. PILCs used in CWPO processes have also demonstrated a great capacity for wastewater treatment, pollutant removal, and even virus inactivation [58, 59]. Among heterogeneous catalysts used in oxidation processes, PILCs are currently of great interest for heterogeneous AOP processes to remove recalcitrant contaminants [60].

**Figure 3:**
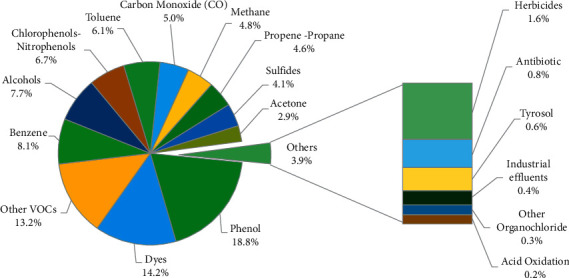
Organic contaminants and CECs removed by advanced oxidation processes employing PILCs as catalysts.

Anthropogenic activities have generated numerous discharges of nonbiodegradable compounds to water bodies. Then, a wide range of catalysts for the oxidation of pollutants, including pillared clays, have been used over the last four decades [61, 62]. Research has mainly focused on the treatment of organic compounds, with particular attention to the removal of emerging pollutants (EPs), also known as contaminants of emerging concern (CECs), in wastewater and drinking water through oxidation processes [63–66]. In the case of AOPs using PILCs as catalysts (AOP/PILC system), the most widely studied pollutants have been substances of difficult biodegradation as shown in Figure 3. In this group of pollutants, phenols, dyes, and VOCs are the main model molecules studied (Top 3). In the case of dyes, these compounds are of particular interest because they inhibit photosynthetic processes in water bodies, are toxic to some aquatic species, and may even affect human health. This is why several authors have reported the removal of azo dyes using PILCs in CWPO processes [50, 67, 68]. Between 1980 and 2019, the most commonly used dyes as model molecules in scientific publications for the treatment of colored wastewater, using AOP/PILC systems, are methylene blue (23%), methyl orange (12%), orange II (12%), rhodamine B (10%), malachite green (7), among others (36%).

Additionally, Figure 3 shows a group of pollutants treated by AOP/PILC systems, including herbicides, antibiotics, medicines, industrial effluents, and organochlorine compounds, part of well-known CECs [69–71]. For instance, herbicides such as 2,4-dichlorophenoxyacetic acid and 2,4-dichlorophenoxypropanoic acid have been removed in 90 min with 80% conversions by photo-oxidation systems and Ti-pillared clays [72]. CWPO processes using Fe/Cu/Al-PILCs are highly efficient in the removal of antibiotic sulfanilamide, achieving removal efficiency of 99% [73]. Similarly, organochloride compounds have been treated through photocatalysis by TiO_2_-pillared fluorine mica, reaching 63% degradation in 30 min and 100% in 5 h, in the treatment of hexachlorocyclohexane (HCH) [74, 75]. As for industrial effluents and persistent organic pollutants, AOP/PILC systems have been extensively studied in the treatment of compounds such as methyl parathion [76], xylenes [77], and coffee wastewater [78]. Equally, some endocrine disruptors such as di-n-butyl phthalate, diethyl phthalate, dimethyl phthalate, and bisphenol-A have been removed through photo-oxidation processes and the use of clay catalysts [79]. This demonstrates that there is a raising trend in research on pillared clays for wastewater treatment towards the removal of organic compounds, CECs, and conventional pollutants. Since this has been a scarcely examined topic, further studies on the removal of pharmaceuticals in wastewater with AOP/PILC systems are required [48].

**Figure 4:**
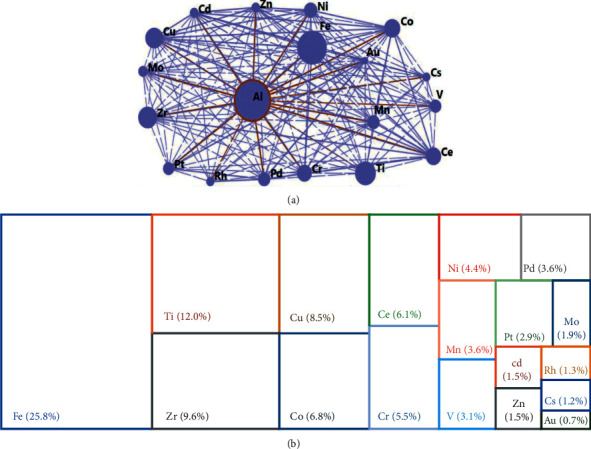
Metals used as active phases in PILCs. (a) Metal network (mixed system) and (b) distribution (%) of supported metals.

Catalytic applications of PILCs in chemical and environmental processes depend on the type of active phases incorporated into them [22, 80]. In the modification of clays through pillarization, different metals have been used for the preparation of pillaring agents [22], being aluminum and iron the two most widely studied metal cations from 1980 to 2019 (Figure 4).  Figure 4(a) shows metals used in the synthesis of PILCs, where the size of the circle is proportional to the number of times the metal was used in the preparation of the pillared clays. The thickness of lines connecting the circles represents the number of times metals have been used together (mixed systems) by co-hydrolysis, impregnation, or post-exchange of a PILC [22].

Aluminum has been the most frequently used metal for the synthesis of metal polycations (Keggin-Al_13_ type) in the modification of clays via pillaring [24, 43]. Although the Keggin-Al_13_ species is the most commonly used pillaring agent, in recent years, Keggin-Al_30_ polycations have also been synthesized [43]. They have been conferred higher thermal stability and more Brønsted acid sites to the PILCs [43, 81]. Nevertheless, both species (Keggin-Al_13_ and Al_30_) form stable pillars after subsequent calcination of the intercalated clay, leading to materials with higher surface area and catalytic activity. Other metals like Fe, Zr, and Cr are potentially capable of acting as pillaring agents, creating stable pillars [82]. Indeed, pillaring agents with two inorganic cations in various molar fractions have been synthesized through the intercalation/pillaring of clay minerals (metal network in Figure 4(a)) to improve thermal, adsorptive, and catalytic properties of the pillared solid [22]. Besides, metal salts such as Al/Fe, La/Al, Fe/Cr, Cr/Al, Fe/Zr, among others can form mixed oxide pillared clays by metal co-hydrolysis [12]. As the size and charge of pillaring species are modified by co-hydrolysis, pillarized clays with mixed systems have more specific catalytic and adsorption properties than single-metal PILCs.

Clay minerals such as montmorillonite can modify their catalytic activity by incorporation of cations such as Al^3+^, Fe^3+^, Cu^2+^, Zn^2+^, Ni^2+^, Co^2+^, and Ti^4+^ [83]. The main active phases supported in PILCs are shown in Figure 4(b). Transition metals most commonly used as active phases in PILCs are Fe and Ti. Particularly, clays modified with the mixed Al/Fe system have shown excellent performance in CWPO systems for the degradation of organic compounds (including contaminants of emerging concern) present in wastewater [84, 85]. Besides, Ti-pillared clays have been particularly used in oxidation processes, and are the most widely studied clay-based materials in photocatalysis [55–57]. Generally, these clays (Ti-PILCs) show a basal reflection of ∼12–25 Å, allowing them to be used in photo-oxidation and adsorption processes of different compounds [86–88]. Even Fe, supported on TiO_2_-pillared montmorillonite for the catalytic oxidation of toluene, has been synthesized [89].

Despite the good results of iron-based materials in heterogeneous advanced oxidation processes for wastewater treatment, the Fe/AOP system shows some disadvantages such as restricted solubility of iron species, high consumption of oxidants, and a low Fe^2+^ production rate. Consequently, other metals with multiple oxidation states and redox stability (Co^2+^/Co^3+^, Cr^3+^/Cr^6+^, Cu^+^/Cu^2+^, Ce^3+^/Ce^4+^, Ru^x+^/Ru^x+1^, and Mn^x+^/Mn^x+1^) have been explored for decomposing H_2_O_2_ to generate •OH radicals in AOPs, even under neutral/alkaline conditions [85]. PILCs with iron-free systems such as Co^2+^ impregnated (Co/Al-PILCs) have been used for the removal of tartrazine with peroxymonosulfate [90]. Furthermore, clays modified by co-hydrolysis of Cr and Al have been synthesized for the oxidation of phenols [91] and carbon monoxide (CO) [92].

Other processes where transition metals have been used in the synthesis of pillared clays or supported on PILCs for catalytic processes were compiled in the literature [24]. Some active phases used with PILCs in catalytic reactions are listed in Table 2.

**Figure 5:**
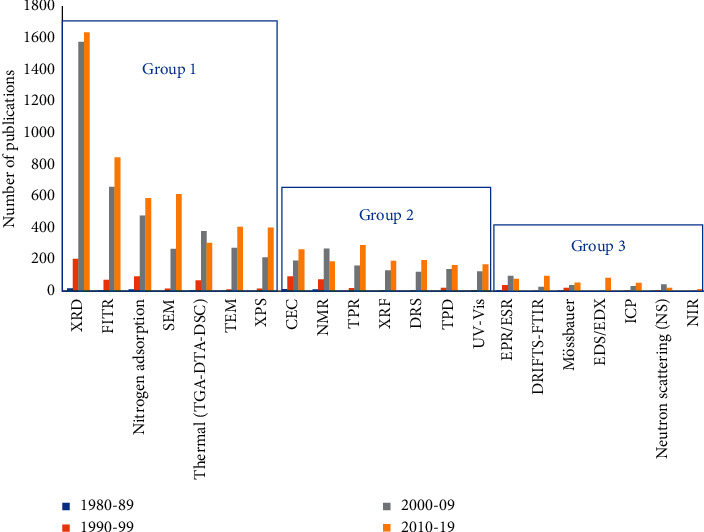
Number of publications that have used specific characterization techniques for PILCs by decades.

Figure 5 shows the evolution of the number of publications that have used different techniques for the characterization of PILCs per decade. These characterization techniques have been applied to both PILCs and pillaring agents [99, 100]. The increase in the number of times that techniques have been used to characterize PILCs per decade is associated with the increase in the number of publications on PILCs during the last 40 years. However, three techniques (NMR, EPR, and NS) show a slight decrease in the number of times they have been applied. The techniques of group 1 have been the most generally used than those belonging to groups 2 and 3.

Group 1 techniques, such as XRD, FTIR, and nitrogen adsorption isotherms, are the most broadly applied since they provide important information on structural, chemical, and textural properties of clays and PILCs [99]. In addition, electron microscopy (TEM and SEM) provides information on the size and morphological variations of clay crystals [101, 102]. Meanwhile, the oxidation state and interactions of active phases supported on PILCs can be obtained by XPS [92]. Thermal analyses (TGA, DTA, and DSC) are used to analyze transformations of polycations and clay minerals due to heating. Similarly, they allow the identification of different stages happening during the thermal transformation of materials [99]. These group 1 techniques belong to the “basic” or “classical” techniques for PILC characterization.

In groups 2 and 3, some spectroscopic techniques can be used in conjunction with other solid-state techniques for the quantitative analysis of PILC characteristics [101, 103, 104]. Less frequently explored techniques such as EPR or Mössbauer spectroscopy provide information of the nature of species formed in pillared solids [105, 106]. Nuclear magnetic resonance allows the study of Al-polycations in pillaring agents and ^27^Al and ^29^Si in clays [107, 108]. XRF has also been used to determine the elementary composition of clays and PILCs [109, 110]. Neutron scattering (NS) provides information on the mobility of water in the intercalary region, while the TPR technique allows to monitor the reducibility of metals in PILCs [99].

Figure 5 also shows a significant increase in the number of times the aforementioned techniques have been used in the last two decades (2000–2019) compared to the 1980–1999 period. This increase is related to a greater number of publications on PILCs since the 2000s.

**Figure 6:**
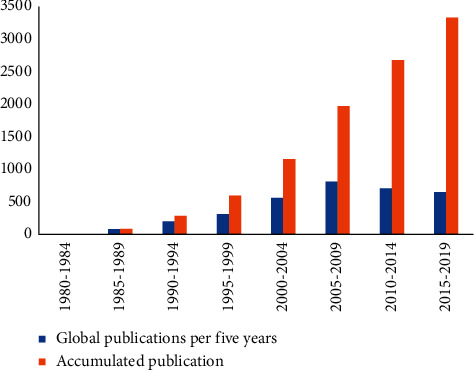
Global publications per 5-year period from 1980 to 2019.

Regarding the evolution in the number of publications per 5-year period, an exponential growth between 1980 and 2009 is shown (Figure 6). However, in the last decade, there is a slight decrease in the number of publications. This behavior can be explained by the bibliometric law of exponential growth [111, 112]. Stages of growth in the publication of scientific literature are explained as follows: the first corresponds to an exponential growth in the number of publications, followed by a linear growth in which its rate is constant. Subsequently, it reaches the saturation limit and ends with the stage of decline in the research field [111]. Publications and authors from 1980 to 1994 are associated with the pioneers of PILC research and the initiation of this scientific area, whereas the period between 1994 and 2009 is related to a possible research front development [113, 114]. However, for the last decade (2010–2019), a slight deceleration in this growth is observed. These fluctuations in the number of publications are normal. Thus, it is premature to associate them with a disinterest of the scientific community in PILCs. Nevertheless, the number of publications should be monitored in the next decades to determine a new growth or decline in the research field [112]. Similar results have been reported in other research areas with fluctuations in the number of publications over the decades analyzed [115].

Despite fluctuations in the number of papers on PILCs, a further increase in the number of publications is expected to occur in the coming years as some researchers have stated the growing interest of the scientific community towards the use of these materials in environmental applications as adsorbents for organic compounds in aqueous solution [43, 116]. Moreover, in the last two decades, the field of application of PILCs has diversified in both catalytic and adsorption processes [48, 117, 118]. Regarding its use as adsorbents, PILCs have been applied for the removal of heavy metals [48, 119] and organic pollutants such as ciprofloxacin [120] and thiabendazole [121].

**Figure 7:**
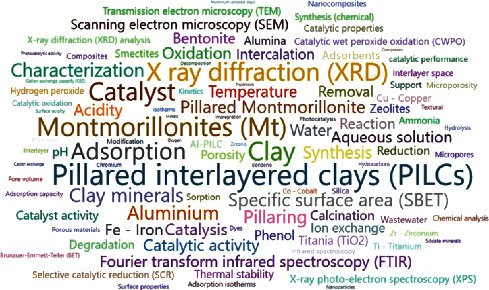
The 100 most commonly used words in publications on PILCs (word cloud).

To identify new trends and research fields in pillared clays, the frequency of keyword usage in scientific publications was quantified through text data mining. The top 100 most frequently used words in PILC publications are shown in Figure 7. The analysis of keywords in the word cloud shows a marked bent towards publications on the characterization of PILCs. The most used techniques, according to the frequency of use of keywords, are XRD, FTIR, nitrogen adsorption, and SEM/TEM. The same trend was identified in Figure 5. Some of these characterization techniques are related to the properties of PILCs, generating a growing interest in its application in catalytic (acidity, catalytic performance/activity, thermal stability, etc.) or adsorption processes (surface properties, sorption capacity, cation exchange capacity, etc.). Although PILCs were initially used in petrochemical processes, Figure 1 shows that their use has decreased in this type of processes and much more in environmental catalysis. Emerging fields in PILCs are mainly related to the application in photocatalytic processes, degradation of pollutants in aqueous media, synthesis of composites, and application in AOPs. Similar trends on the approach towards the use of PILCs in green chemistry were described in the first decade of the 2000s [17]. Thus, these research trends have been maintained constant in the last two decades.

From the bibliometric analysis, it was also identified that, in Scopus, research areas with the greatest interest in PILCs are chemistry, chemical engineering, materials science, and environmental sciences, whereas in WoS, they are chemistry, engineering, materials science, and environmental sciences. This trend demonstrates a growing interest in pillared clays among the scientific community through different areas of research/knowledge.

**Figure 8:**
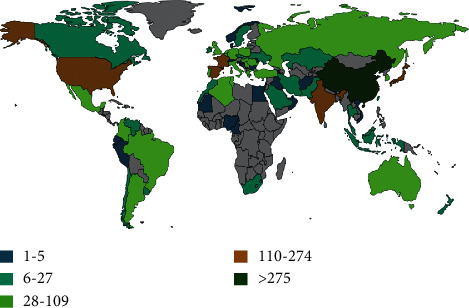
Number of publications on PILCs by country.

The distribution of countries that have published on PILCs is shown in Figure 8. The five countries with the highest number of documents published on the subject are China with 32% of the world publications, followed by Spain with 15%, the United States 14%, India 11%, and France 7%. Through bibliometric analysis and text data mining, it was determined that the countries with the most publications in international cooperation are Australia, France, Belgium, Brazil, Algeria, Spain, and the United States. One of the regions that has shown particular interest in pillared clays in the last two decades is Ibero-America. However, the continents with the highest number of publications on the subject are Asia and Europe.

**Figure 9:**
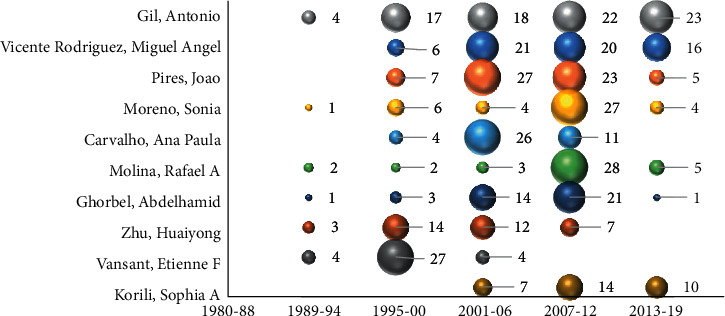
The top 10 authors who have published the most on PILCs (timeline).

The top 10 authors publishing the most on PILCs (Figure 9) are, in order of highest to lowest scientific production, as follows: Antonio Gil (*h*-index 42), Miguel Á. Vicente (*h*-index 39), João Pires (*h*-index 37), Sonia Moreno (*h*-index 30), Ana Paula Carvalho (*h*-index 39), Rafael A. Molina (*h*-index 31), Abdelhamid Ghorbel (*h*-index 29), Huai Yong Zhu (*h*-index 67), Etienne F. Vansant (*h*-index 55), and Sophia A. Korili (*h*-index 28). It was identified that authors with the highest number of publications on PILCs do not correspond to those with the highest *h*-index. This is because many of the authors work in different lines of research, diversifying their scientific work and, therefore, being cited for scientific publications unrelated to PILCs, thus increasing their *h*-index.

**Figure 10:**
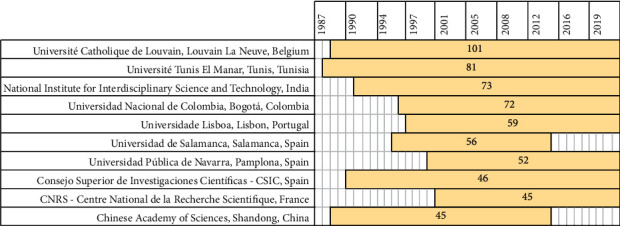
Timeline of the top 10 institutions with the highest number of publications.

Seven of these authors are linked to Ibero-American institutions and the remaining three to Tunisia, Australia, and Belgium (Figure 10). Although China is the country with the highest number of publications on PILCs, none of its authors is in the top 10. It was also determined from Lotka's law that this small group of authors (top 10) publish the largest number of articles being the most representative and productive on PILCs. Nevertheless, in the last two decades, new authors have been emerging in this area of study [122].

It is observed in Figure 9 that the highest number of publications by the authors in the top 10 occurred at the end of the 1990s and the first decade of the 2000s. In addition, the timeline shows that none of the authors had publications in the period 1980–1988, so they are not part of the group of pioneer researchers and, most of them, have decreased the number of publications in the last decade. Although the top 10 authors have been highly cited, none of them are authors of the most cited articles on PILCs, which is summarized in Table 3.

Articles in Table 3 are considered “classics” and are mostly review articles with some of their authors being precursors of PILC research, such as Dr. Thomas J. Pinnavaia. The most cited articles belong to the following journals: Science, Applied Clay Science, Advances in Colloid and Interface Science, Catalysis Today, Nature, Catalysis Reviews-Science and Engineering, and Tetrahedron. However, journals that publish the most on PILCs are as follows: Applied Clay Science with 160 publications, Microporous and Mesoporous Materials (86), Applied Catalysis A: General (83), Catalysis Today (80), and Applied Catalysis B: Environmental (72). From this group, only two journals have published from the 1980s until 2019 (Applied Clay Science and Catalysis Today) while the other started their publications by the 1990s.

According to Bradford's law, it was determined that only 11 journals (corresponding to 2% of the total) publish 32% of the articles on PILCs. Therefore, these journals are considered to be the most specialized on the subject [131].

The same behavior observed in the journals occurs with the 10 institutions that publish the most on PILCs (Figure 10). Only four institutions started their research in the 1980s and the rest in the 1990s. Most of these institutions continue publishing on PILCs and only two of them do not report any associated publications in the last 5 years (University of Lisbon and Catholic University of Louvain). It was also observed that five of the 10 institutions belong to Ibero-American countries (Spain, Portugal, and Colombia), demonstrating the interest of the region towards the use of PILCs in catalytic processes, particularly in AOPs [4].

## 4. Implications

The results of this bibliometric analysis have implications for the scientific community working on the synthesis and characterization of layered solids, such as clays, which can take advantage of the information contained in this article and dataset. The bibliometric analysis permitted the identification of the most important applications of PILCs and it provides valuable information on the evolution of scientific production in this field over the last four decades.

This article analyzed a large number of studies that have been published on PILCs identifying their uses as adsorbent materials and catalysts/supports of transition metals in heterogeneous catalysis. Particularly, this study focused on the application of PILCs for water treatment through advanced oxidation processes (AOPs) in the removal of organic pollutants. In addition, this BA determined that PILCs have been used in petrochemical reactions and chemical and industrial processes, which shows the versatility and usefulness of these materials in scientific research.

The dataset (supplementary material) also allows a scientific mapping such as cooperation of authors and institutions publishing the most on pillared clays in different countries (co-authorship networks). Furthermore, it is possible to identify frequently used keywords, areas of major interest on PILCs, characterization, and synthesis techniques most commonly employed in studies on pillared clays.

## 5. Conclusions

In the last four decades, pillared interlayered clays (PILCs) have been widely applied in chemical processes. However, the last two decades have seen an increasing interest towards applications in environmental catalysis, especially in advanced oxidation processes. In particular, PILCs have been used in photo-oxidation and CWPO processes for the treatment of organic pollutants and CECs.

The most highly used metal for the synthesis of a pillaring agent is aluminum (Al) due to the formation of large polymeric species (Al_13_) that create stable pillars after calcination. Additionally, the most supported active phases in PILCs are Fe, Ti, Zr, Cu, Co, and Ce. The main application of Fe and Al active phases in PILCs favors Fenton-like and photocatalysis processes.

From 1980 to 2019, the mostly used techniques for the characterization of PILCs were XRD, FTIR, nitrogen adsorption, thermal analysis, SEM/TEM, and XPS. These techniques have been used for the chemical, textural, and structural characterization of pillared clays. Likewise, spectrophotometric techniques such as NMR, UV-Vis, Mössbauer, EPR, DRIFTS-FTIR, among others have been used for the characterization of pillaring agents, pillars, and the surface of clays/PILCs.

The number of publications has grown in recent decades, particularly in the first decade of the 2000s. However, a slight decrease is observed from 2010 to 2019. This deceleration in the number of publications is a normal fluctuation. An increase in publications is expected, though, due to the versatility of synthesis (modification), wide range of applications, and growing scientific interest in PILCs.

The countries with the highest number of publications are China, Spain, France, the United States of America, India, and Japan. Ibero-America is one of the worldwide leading regions in research on PILCs, with some of its authors and institutions in the global top 5 and 10.

The most referenced publications on PILCs are review articles. Journals publishing the most on pillared clays are Applied Clay Science, Catalysis Today, and Applied Catalysis B: Environmental and all are considered high-impact journals.

## Figures and Tables

**Table 1:** Search strategy for retrieval of documents in Scopus and Web of Science (WoS) databases from 1980 to 2019.

Item	Description	
Keywords	“Pillared clays” or PILCs or PILC or “pillared interlayered clays”	
Core collection	Scopus	Web of Science (WoS)
	Not applicable	Science citation index SCI Emerging sources citation index ESCI Note: social science, arts, and humanities collections were not included
Document type	Research articles, reviews, and book chapters	
Subject area	Environmental science, chemistry, chemical engineering, engineering, materials science, multidisciplinary, Earth, and planetary science	Geochemistry, geophysics, geology, chemistry, materials science, crystallography, engineering, mineralogy, environmental sciences, ecology
Limited by	Title	Title
	Abstract	Abstract
	Keywords	Keywords
		KeyWords Plus^a^
Records	1986	1439
Total records	3425	
Combined records	2511	

^a^ KeyWords Plus are words or phrases derived from the titles of the references in the publications [38].

**Table 2:** Pillared clays and active phases used in chemical and/or environmental processes.

Metal/PILC	Catalytic application	Ref.
Fe, Cu, Mn	Catalytic degradation of organic pollutants	[58]
Pt, Pd, Rh	Hydrodechlorination of chlorophenols	[93]
Cu	Oxidation of toluene through CWPO process	[94]
V	Oxidation of hydrogen sulfide	[95]
Fe, Zn	Oxidation of orange II	[96]
Ni	Electrochemical oxidation of phenol	[97]
Co	Oxidation of sunset yellow using the bicarbonate-activated hydrogen peroxide system	[98]

**Table 3:** Most frequently cited articles on PILCs in the last four decades.

Number of citations	Title	Ref.
1507	Intercalated clay catalysts	[123]
845	Traditional and new applications for kaolin, smectite, and palygorskite: A general overview	[124]
823	Adsorption of a few heavy metals on natural and modified kaolinite and montmorillonite: A review	[125]
703	Preparation and catalytic properties of cationic and anionic clays	[126]
639	Assembly of porphyrin building blocks into network structures with large channels	[127]
493	Clays and oxide minerals as catalysts and nanocatalysts in Fenton-like reactions - A review	[128]
466	Pillared clays as catalysts	[129]
476	Clay and clay-supported reagents in organic synthesis	[130]

## Data Availability

Data supporting the analysis in this research article were included in the form of tables and figures. The dataset used to support the findings of this study have been deposited in the Mendeley Data repository (DOI: 10.17632/s44bj88rx2.1).

## Supplementary Materials

Supplementary Materials Supplementary data correspond to metadata retrieved from the main databases (Scopus and WoS) of scientific publications on pillared clays (PILCs) used in chemical and environmental processes from 1980 to 2019. .
