# Supplementary material for: Research Trends on Pillared Interlayered Clays (PILCs) Used as Catalysts in Environmental and Chemical Processes: Bibliometric Analysis
Source: ScientificWorldJournal. 2022 Mar 2;2022:5728678. doi: 10.1155/2022/5728678 (PMC8906976; doi:10.1155/2022/5728678)
Supplement: Supplementary Materials — Supplementary data correspond to metadata retrieved from the main databases (Scopus and WoS) of scientific publications on pillared clays (PILCs) used in chemical and environmental processes from 1980 to 2019. . [file 5728678.f1.docx]

**Supplementary material**

Datasets generated during the current study are available in the Mendeley Data repository, found online at <https://data.mendeley.com/datasets/s44bj88rx2/1>

Supplementary data correspond to metadata retrieved from the main databases (Scopus and WoS) of scientific publications on pillared clays (PILCs) used in chemical and environmental processes from 1980 to 2019. These data allow visualizing research trends on the use of PILCs in catalytic processes over the last 40 years. The file named "PILCs 1980-2019.csv or PILCs 1980-2019.txt or PILCs 1980-2019.xml" allows text and data mining on the scientific productions of pillared clays.

***Cite this dataset***

Macías-Quiroga, Iván Fernando; Rengifo-Herrera, Julián Andrés; Arredondo-López, Sandra Milena; Marín-Flórez, Alexander; Sanabria-González, Nancy Rocío (2021), “Bibliometric dataset of pillared clays (PILCs) as retrieved from Scopus and Web of Science databases: research trends”, Mendeley Data, V1, doi: 10.17632/s44bj88rx2.1
